# The Tale of Asbestos in Sweden 1972–1986—The Pathway to a Near-Total Ban

**DOI:** 10.3390/ijerph14111433

**Published:** 2017-11-22

**Authors:** Peter Westerholm, Bertil Remaéus, Magnus Svartengren

**Affiliations:** 1 Department of Medical Sciences—Occupational and Environmental Medicine, Uppsala University, 75185 Uppsala, Sweden; magnus.svartengren@medsci.uu.se; 2 Private Residence, Solängsvägen 28, 192 54 Sollentuna, Sweden; remaeus@hotmail.se

**Keywords:** asbestos, asbestosis, lung cancer, mesothelioma, occupational hazard, public-health hazard, disease prevention

## Abstract

This paper provides a narrative of the national intervention strategy in Sweden aimed to restrict the industrial use of asbestos. For many years, asbestos was imported for widespread industrial use, resulting in large amounts throughout Swedish society. In 1972, the whistle was blown in a Communist Party parliamentary motion describing asbestos as a health hazard and requesting action to prohibit its use. Although the motion was rejected, it initiated the extensive charting of asbestos sources on a tripartite basis, involving government agencies, and employer and trade-union organizations. Restrictive asbestos management practices were enforced from July 1982. The year 1985 saw the Government Asbestos Commission review, covering use-determining factors, international regulations, and assessments of cancer risks. The relative risks of chrysotile and amphibole were considered internationally (by the IARC), since chrysotile (a Canadian export) was regarded as unharmful in Canada at that time. Prohibiting asbestos use resulted in its virtual disappearance as an import to Sweden from the early 1980s. However, asbestos has undergone a transition from an occupational to a public-health hazard (although some work-related hazards, such as handling and disposal, remain). The transition reflects the public’s exposure to existing stocks, in homes, workplaces, etc. Mesothelioma incidence has come to be regarded as an indicator of prevention effectiveness.

## 1. Introduction

We give an overview of changes introduced in Sweden during the 1970s and 1980s into the regulation and use of asbestos as a raw material. The changes concern its use in industry, buildings, water pipes, friction materials installed in motor vehicles, trains, and ships, ventilation systems of many types, and a broad variety of consumption goods. These changes had points of departure primarily in aspects of occupational health related to the exposure of workers to airborne asbestos fibres. There is a chain of work processes involved, starting with asbestos mining and extending to the end use of manufactured products and the management of asbestos waste.

The changes were made for a variety of reasons, and there was a downgrading of asbestos use resulting from successive legal regulations restrictions and supervision from the mid-1970s onwards. The downgrading process in Sweden was brought to a provisional end by the adoption in December 1981 by the Swedish Working Environment Agency of revised directives on the management of exposure to asbestos fibres [1]. The directives were collectively called “Asbest” in Swedish, and were to be enforced from 1 July 1982.

The date 1 July 1982 marked a new start for the comprehensive management of workers’ exposure to asbestos that also included aspects of public health. The hazard assessments of occupational exposures to asbestos fibres were radically reviewed at multiple levels, including the Parliament, the Department of Labour (politically), employer and trade-union organizations, and the National Board of Occupational Safety and Health (NBOSH) and Chemical Safety. The media and academia were also involved and, ultimately, employers in collaboration with their employees. Tumours of the lungs and highly malignant tumours in tissues of the pleural and peritoneal sacs (mesothelioma) were identified as critical outcomes related to exposure to asbestos. Earlier, the critical preventive health outcome had been lung asbestosis. Although asbestosis of the lungs was still to remain on the list of targets for prevention, the revised hazard assessments were to have significant consequences for the enforcement of the Working Environment Act and for collective agreements in the labour market between employers’ organizations and trade unions. 

In this paper, the focus is on the development of hazard assessment and management of occupational exposure to asbestos fibres during the 1970s when there was a transition to more restrictive practices in the use of asbestos materials and in the management of exposure to asbestos fibres in the workplace to include wider issues of public health. This implies a shift in attention to the issues involved in the management of asbestos exposures resulting from the investment in asbestos materials installed and used for many years in the past. It is not to be expected that these issues can be resolved simply by banning the present-time use of asbestos. 

## 2. Background and Definitions

**Figure 1:**
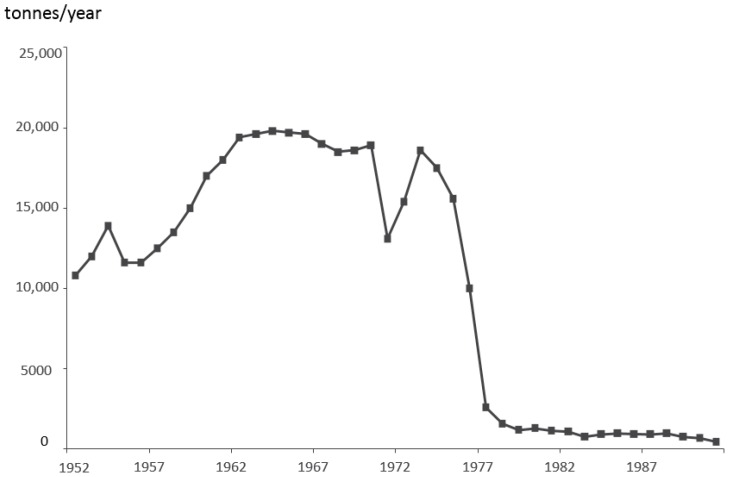
Imports of raw asbestos to Sweden, 1952–1991.

In this paper, the term “asbestos” is used to denote mixtures of fibrous forms of silicate minerals crystallized to form long and thin fibres mined and processed as raw materials, and sold under the commercial generic term asbestos. The mining of asbestos during the years 1965–1975 occurred mainly in exporting countries: Russia (the former Soviet Union), the People’s Republic of China; the United States; Brazil; Canada; Cyprus; the Balkan states of former Yugoslavia; and Australia. Estimates of this production was 95% chrysotile and 5% amosite or crocidolite asbestos. The worldwide production of asbestos in the years 1968–1978 has been estimated (in thousands of tonnes) as: 3291 (1968), 4598 (1973), and 5818 (1978). Imports of raw asbestos/asbestos products to Sweden (in thousands of tonnes) have been estimated as: 16.7/15.9 (1968); 18.7/14.8 (1978); 1.3/4.5 (1978), and 1.0/1.4 (1984). Imports of asbestos to Sweden from 1952 to 1991 are shown in Figure 1 ([2,3], p. 877, reused with the authors’ consent). There was a dip in the late 1960s, followed by a more permanent and rapid fall, reflecting the prohibition of asbestos spraying, from 1973 onwards.

The types of asbestos used in Sweden were: chrysotile (the serpentine form of asbestos); the amphibole forms, crocidolite or blue asbestos; amosite or brown asbestos; tremolite; and anthophyllite and actinolite. Due to their valued properties, asbestos and fibre products have, from early on, been extensively used in many applications:

- Thermal insulation—pressurized vessels and plumbing for railways, ships, and maritime transport vessels.
- Noise insulation—acoustic insulation sheets.
- Mechanically resistant material in packaging, brake and clutch linings, filters, and gaskets in road vehicles, and textiles and fabrics.
- Protection against condensation in ventilation systems and pipelines.
- Fire hazard protection—spraying of asbestos fibres onto steel constructions, ventilation systems, firewalls, etc.
- Concrete construction material in supportive building structures.
- Supportive texture and quality in paints, glues, etc.

The use of asbestos has been assessed through Swedish statistics on the registered imports of raw asbestos (Figure 1). Although the data cover all mineral forms of asbestos, 95% was chrysotile. Imports of asbestos to Sweden accelerated after World War II, but levelled off in the 1950s. As seen in Figure 1, there was a near-disappearance of asbestos imports from the late 1970s. The health effects attributed to exposure to asbestos, caused by the inhalation of respirable fibres were, at the outset of the 1970s, observed in target body organs, lungs, and airways. They depended on exposure doses and characteristics. The pathological changes and associated disease entities were:

- Asbestosis (lung fibrosis), commonly classified as a type of pneumoconiosis. Occupational exposures to levels of asbestos fibres above threshold limit values are seen as possibly causing lung asbestosis that affects lung function.
- Pleural plaques. Hyaline bodies formed in the pleural sac, are commonly regarded as benign, not affecting lung function.
- Pleurisy—the effusion of body fluid into the pleural sac. It is commonly symptomless, but may cause discomfort and pain, dyspnoea, and fever. Pleurisy may possibly be a precursor to fibrotic changes involving the pleural sheaths and lung tissue.
- Malignant tumours in airways and lung tissue—lung cancer.
- Malignant tumours in pleural mesothelial or peritoneal tissues—mesothelioma.

There were early signs of cancer risk induced by exposure to asbestos fibres in reports published in 1935 cases of lung cancer in patients with asbestosis by Gloyne [4] and by Lynch and Smith [5] (see the IARC Monograph Asbestos 100 C (Chrysotile, Amosite, Crocidolite, Tremolite, Actinolite and Anthophyllite) [6]). The work of Dr. Gloyne and Dr. Merewether in the 1930s is described in readings from WHO’s Pan American Health Organization [7]. It is noteworthy that this is almost 80 years ago, and asbestos is still widely used, especially in Asia. The first cohort study demonstrating an excess of lung cancer among asbestos-exposed workers was of textile workers by Doll in 1955 [8]. Since 1955, an association between lung cancer and occupational exposure to asbestos has been demonstrated in numerous epidemiologic studies.

The first report of a possible association between asbestos exposure and mesothelioma was published by Wagner and colleagues in 1960 ([9], pp. 3–7). It described an outbreak of mesothelioma in a crocidolite mining region in South Africa. The causal association between asbestos and mesothelioma has since been well-established in numerous research reports. 

In the enforcement of legislation in Sweden, the critical body organs and health outcomes related to exposure to asbestos were, until 1950–1970, the lungs and pleural sheaths. The outcome most commonly referred to was “asbestosis”. There was, at this time, however, no official consensus on that critical outcome, nor any commitment to the prevention of malignant tumours of the lungs and pleural sheaths caused by asbestos exposure. With regard to other cancers associated with asbestos exposure, the scientific literature is sparse. It is, however, important to note that considerable attention has been paid to cancers of the ovaries, larynx, pharynx, and the gastrointestinal tract, including the oesophagus and colorectal tract. There are also other, unconfirmed locations. Thus, the lungs and pleural sheaths are not the only possible cancer endpoints. 

## 3. The Reappraisal of Hazard Assessments and Developments from 1972 Onwards

In January 1972, a group of Swedish parliamentarians representing the Communist Party submitted a motion proposing the adoption of a law prohibiting the use of asbestos. In its summary, the motion made reference to “occupational exposure to fibres of asbestos representing a risk of contracting occupational cancer”. The tabled motion finished with the statement: “In our view, asbestos as a raw material may be substituted in many, perhaps in even all, of its current applications. Taking into account the steady increase in asbestos imports, and also the use of asbestos despite the availability of substitute materials, and the health hazards of exposure to asbestos fibres having been recognized and well-documented for many decades, a categoric prohibition of the use of asbestos seems to us to be more than well-founded. … We, John Takman, Sune Olsson, Lars-Ove Hagberg, Karin Nordlander, John Magnusson, Lars Werner, Karl Hallgren and Per Israelsson, propose that the (Swedish) Parliament requests the Government to present a draft proposal for a law text clearly prohibiting all use of asbestos” [10].

In this parliamentary motion references were made to an article published in the Journal of the Swedish Medical Association by Nils Ringertz [11], where detailed references were made to studies published in the UK, the US, Austria, France, and Germany, indicating that lung asbestosis was associated with an increased risk of lung cancer, and also with the relatively rare, but most malignant tumour disease, mesothelioma of the pleura and peritoneum. Reference was also made to the “Environment” scientific journal’s issue of March 1969, which contained a critical review by Dr. Irving Selikoff, from the US, of health outcomes associated with asbestos exposure in shipyards [12]. 

The motion by John Takman, with eight signatories, was processed according to the standard parliamentary procedure of the time. This entailed extensive review by public authorities, research institutions, labour-market organizations and social partners, and non-governmental organizations. This processing of the motion was documented in the Parliament as Report No. 1972:40 of the Health and Social Commission [13].

The cat was out of the bag. An extensive process had been set in motion. 

The Swedish Parliament ultimately declined to follow the Takman recommendation, but the deliberations of the Swedish Parliament triggered an intense debate that was followed closely by the news media and the public. At the political level, there was general agreement over the relevance of the issues raised in Motion 1972:480. In Parliament, there was near total agreement on the need to undertake a thorough review of current occupational safety and health regulations with particular regard to issues of exposure to asbestos fibres.

The total prohibition of asbestos, as recommended, however, received only minority support in the Social Affairs and Safety and Health Commission of the Parliament. In the review of issues pertinent to the proposed prohibition of asbestos, many of the organizations involved had reservations about recommending the elimination of asbestos when suitable raw materials were not available. The necessity of reviewing substitute raw materials and production techniques, for both health and safety reasons and effectiveness/rationality in industrial production, was obvious to all involved.

The decision of the Parliament and the Government was to refer the matter to government executive organs, namely NBOSH and Chemical Safety. To this decision were added a recognition of the problems addressed in the motion tabled by John Takman and associates, and a recommendation of follow-up action to be taken by the Government. In the Parliament, there was a general preference for managing asbestos health hazards by taking restrictive measures in combination with public surveillance of action to be taken by stakeholder organizations in the labour market. In the construction industry branch, this implied forging an immediate consensual agreement between employers and trade unions to establish joint involvement in the reviews to be undertaken by NBOSH of all matters related to asbestos management on a tripartite basis. This new procedure was adopted without delay by NBOSH. It was consistently observed from 1975 to 1978, resulting in a series of new or revised regulations/guidelines. The following subjects were addressed:

- The measurement of asbestos-fibre dust.
- Review of the use of crocidolite (the blue-asbestos mineral).
- Respiratory protection equipment.
- Spraying of asbestos material in buildings for fire protection.
- Use of carpets clad with textile fabrics.
- Asbestos in wall painting materials, glues, putties and fillers.
- Asbestos in the handling or sawing of pre-build structures with asbestos or pipelines of asbestos cement.
- Asbestos in floors and wall claddings.
- Asbestos in ventilation systems (fire protection).
- Demolition, reconstruction or repair of buildings containing asbestos.

In September 1975, a field study was submitted to the National Board of Health and Welfare by Dr. Anders Englund, at the time Medical Advisor to the Swedish Trade Union Confederation (LO), presenting an account of the occupations of subjects registered in the Board’s National Cancer Register [14]. This study had been initiated earlier by Dr. Englund in 1969 when employed at the National Institute of Public Health/Institute of Occupational Medicine. In earlier preliminary analyses at the National Cancer Registry, an aggregation of mesothelioma cases had been observed in persons previously employed in building and construction. At the 1975 presentation, this observation was qualified by the further observation of an aggregation of mesothelioma deaths among former employees of a factory for the manufacturing of railway locomotives (NOHAB Inc., Trollhättan, Sweden). Here, crocidolite (blue asbestos) had been abundantly used for heat insulation. The newly-presented observation received extensive media attention, adding considerable weight to the ongoing debate and putting pressure on government authorities and public-health agencies in the years 1973–1975 to review regulations, hazard assessments, guidelines, and current practices in the management of workplace hazards, in general, and of products containing asbestos, in particular.

The use of crocidolite was banned by NBOSH in 1973. The rapid decline in imports of asbestos during the first half of the 1970s was, however, mainly due to an agreement between employers and unions, acting on a tripartite basis with NBOSH, to abandon the use of asbestos-containing products and to opt for alternative materials. Such tripartite involvement at the national level in the management of work with asbestos was adopted without delay by NBOSH for the entire labour market, including in the services sector. At this stage, the occupational health and safety (OSH) organization of the building industry had access to its own county-based units, “Bygghälsan” (the “Swedish Building and Construction Industry Health Services”). This turned out to be a valuable asset, particularly given that the issue of asbestos-cement material was one of the first to be addressed in policies agreed in consensus.

This new form of collaboration between NBOSH and the partners to the labour market became instrumental in preparing the review of the asbestos regulations of 1981 by considering the practical implications of reappraising the hazards involved (for a full list of appraisals over the period we are considering, see Appendix A). This revised regulation, the “Asbestos Directive 1981:23”, had three principal clauses: (1) material with an asbestos content was to be used only with authorization granted by NBOSH; (2) buildings with an asbestos content were to be repaired or maintained with such material only as permitted by the Labour Inspectorate, under the condition that less hazardous material was not available; and (3) there should be detailed regulation of work involving the repair and demolition of buildings and constructions with asbestos materials.

The first general clause on the restriction and the authorization of exemptions was later qualified following review by the Government Asbestos Commission 1985 [15,16]. In its reports, it considered the issues involved in prohibition of the use of asbestos products in 1986, unless authorized as above, or, optionally, unless a notification of working with asbestos was submitted to the Factory Inspectorate (the regional organization of NBOSH). Exemptions would depend on the size and scope of the project to be undertaken. 

The second general clause included an obligation for all asbestos work to be subject to careful planning with a view to keeping exposure to asbestos dust at the lowest achievable level for those carrying out work tasks, and for all others working nearby.

The third general clause of NBOSH Directive 1981:23 addressed asbestos work involving the demolition and repair of buildings or technical equipment with an asbestos content. In all such work, the staff safety delegates of the enterprise in charge were to be informed in advance, and a written notification of the project was to be submitted to the Factory Inspectorate.

Following the prohibition of import, supply and use of asbestos, and improved working conditions resulting from active workplace prevention programmes from the mid-1970s onwards, the exposure of workers to asbestos fibres has been successively decreased, or even eliminated. However, building maintenance workers and tradespeople can still remain at significant risk if unknowingly exposed to dust from asbestos previously installed in buildings.

The brief of the Government Asbestos Commission of 1985 was to review existing sources of exposure of workers and the general public to asbestos fibres in both occupational and non-occupational settings. Professionally competent groups were assembled to focus on the following sectors: (1) friction materials including brake linings of road vehicles; (2) asbestos used in buildings and in ventilation systems, and for fire protection in trains, ships, and maritime transport systems; (3) other uses of asbestos in industry; (4) asbestos used in water supply and sewage systems and pipelines; the management and depositing of asbestos waste; and, asbestos in the external environment; and (5) health services and health surveillance.

In setting up the Asbestos Commission, the Government also charged Sweden’s National Board of Trade with the task of initiating a notification within the GATT and EFTA international organizations. This was in preparation for the prohibition of the import and sale of road vehicles and motorcycles with brake systems containing asbestos with a view to enforcement from 1 January 1987.

According to the Asbestos Commission, it was not feasible to provide detailed guidelines for the management of the numerous applications and uses of asbestos. In a quick initial review of asbestos applications in the mid-1970s, roughly 3000 different ones were identified. The basic principle applied by the Commission was that there was an explicit expectation for action to be taken by all government agencies and organizations following the instructions and mandates that they had already received. 

Upcoming issues were to be brought back for further consultation with the Government.

The reports of the Asbestos Commission initiated action in the following domains: inventory of the asbestos content of ventilation systems in buildings; demolition/repair of buildings with asbestos; training programmes for staff deployed in asbestos work; review of regulations for the labelling of products with an asbestos content; sanitation programmes for railway wagons, ships, and maritime vessels; and for packing materials and gaskets in printing machines, elevators and industrial machinery with an asbestos content.

The reports of the Commission were to be promulgated and further enforced through detailed directives and guidelines for the management of asbestos work and products with an asbestos content. In the reports, actors were informed of an extension of the primary task of preventing occupational exposure to asbestos dust to measures also to address non-occupational exposure, where any citizen could be exposed without being aware of it.

In this process, which stretched from 1972 well into the first decade of the 21st century, a great deal was learned about the subject of asbestos. Asbestos may be particularly difficult to identify, locate, and assess, and also to evaluate with regard to the potential likelihood of fibres being released. All this applies both to domestically purchased or imported goods, and to earlier building constructions. We also have experiences of attempts to remove asbestos from structures where it is firmly grounded, leading to the emission of fibres and generating a health hazard in places where there was earlier no risk.

## 4. Summarizing the Tale of Asbestos in Sweden between 1972 and 1986

In sum, the story of asbestos in Sweden between 1972 and 1981 may be seen as consisting of seven partly-overlapping phases or time periods:

1972. Whistle-blowing. Parliamentary Motion no. 1972:480, tabled by John Takman MP and colleagues in January 1972, requesting steps to be taken to prohibit all use of asbestos. The motion triggered a thorough process of parliamentary examination and a detailed report presenting the broad issues to be addressed. The motion was ultimately defeated in Parliament. It had, however, set the stage for the framing of a public and political debate to address health aspects of exposure to asbestos fibres.

1973 onwards. Charting of sources of exposure. Increasing general awareness of the importance of knowing the location of asbestos materials in buildings and products, and the extent to which they can release fibres to be inhaled by workers or members of the public.

1974. A powerful media-induced signal. Reappraisal of the health hazards and outcomes involved. A triggering event in the form of intense media attention paid to mesothelioma deaths among previous workers at a locomotive factory in Southern Sweden. Use of crocidolite asbestos for steam boiler insulation in manufacturing suggested as the cause. Ensuing debate added to pressures for action to be taken by government agencies and market stakeholders. Additionally, significant impetus to the idea that mesothelioma should be seen as a critical health outcome in its own right, as well as lung asbestosis.

1974 onwards. Seeking of consensus among government agencies and non-governmental organizations (NGOs). Collaboration of government agencies primarily in the domain of occupational safety and health with employer and trade-union organizations (NGOs) over the identification of hazardous sources and the finding of consensual solutions in relation to difficulties and obstacles to overcome.

1975–1981. Asbestos hazard reappraisal. Acceptance of the principle of regarding all forms of asbestos fibres as potentially hazardous and tumorigenic. Risk of mesothelioma regarded as a critical outcome and determinant of hazard perception, superseding asbestosis in lung tissue. Non-occupational exposure to asbestos fibres were increasingly taken into account. 

At the same time, awareness of asbestos as a material bringing benefits to society, associated with its valuable properties of being technically strong and resilient in many regards, while being a source of fibres which, when inhaled, may cause fatal disease to workers and to others (see also Appendix A).

1981–1986. Setting the stage for joint action. Government agencies, public-health stakeholders, communities, schools, workers, employers and enterprises in branches of industry, communities, schools, and society at large to jointly achieving balanced approaches to the management of exposure to asbestos fibres.

1986 onwards. Revisiting of asbestos management by the Government’s Asbestos Commission. The Commission to take on a broader range of issues, including: many non-occupational sources of exposure; regulatory systems for chemical safety, fire-hazard protection, and road and railway safety; schools and other public buildings, universities, and research establishments; health service establishments, hospitals, asbestos waste management, and disposal.

The initiatives of the Asbestos Commission have meant continuously entering into new domains for the exploration of health issues related to asbestos fibres in the external environment, in the supply and sanitation of tap water and in consumption goods of many kinds, leading to contact and interaction with a broad range of societal and private sectoral and industrial organs. The process is ongoing.

## 5. An International Perspective: The Role of the International Agency for Research on Cancer (IARC)

The IARC (Lyon, France), a UN/WHO agency, has continuously monitored the development of scientific assessments of the carcinogenicity of all forms of asbestos fibres. A summary IARC statement from 2009 was explicit: “There is sufficient evidence in humans for the carcinogenicity of all forms of asbestos (chrysotile, crocidolite, amosite, tremolite, actinolite and anthophyllite)”. This implies that: “All forms of asbestos, as referred to within parenthesis in the text above, are carcinogenic to humans (Group 1)” [17].

This statement on the same subject in an earlier IARC monograph from 1973 had been phrased more generally: “In manufacturing and application industries mesotheliomas have been caused by exposure to crocidolite, and less frequently to amosite and chrysotile”. 

The difficulties in making reliable assessments of risk differentials when comparing different forms of asbestos were recognized early on in the international scientific community. In studies and meta-analyses published later by Hodgson and Darnton in 2000 [18] and by Berman and Crump in 2008 [19], estimates of ratios for mesothelioma were 1, 100, and 500 for chrysotile, amosite, and crocidolite, respectively.

These estimates of asbestos risk differentials regarding the mesothelioma hazard have not implied any changes to the hazard evaluations of governmental agencies or NGOs in Sweden. 

## 6. Mesothelioma and Projections of Its Incidence in Sweden

During the years 1975–1985 mesothelioma has become a critical outcome in assessments of asbestos hazard outcome effects. Critical in the present context means that it is a determinant of action taken by stakeholders. Mesothelioma has properties that need to be taken into account with regard to prevention:

- It is an aggressive malignant disease commonly causing death of the subject within one year of clinical diagnosis.
- Mesothelioma’s crude annual incidence (M + W) in Sweden was between three and four per 100,000 population in the years 2011 through 2015. By comparison, the crude lung cancer annual incidence in Sweden (M + W) was between 80 and 85 per 100,000 population in the same years.
- The time from exposure to the hazardous agent and manifest mesothelioma is often 35–40 years or more.
- Many people contracting mesothelioma have no known exposure to asbestos fibres.
- Sweden observed a decrease in mesothelioma incidence due to restrictions on the use of asbestos in the years 1974–1981. Järvholm, Englund, and Albin [20] started a follow-up in 1999, noting that a longer period of post-intervention observation was needed for valid testing of the hypothesis that the decrease in incidence was caused by the ban on asbestos that became effective in 1975. The extent of decline in the mesothelioma risk was regarded by Järvholm and Burdorf in 2015 [3] as primarily to be observed in generations of young workers who started their working careers during or after introduction of the restrictions. Swedish cancer statistics (from the National Board of Health and Welfare) present a seemingly unchanged crude number of nationally-observed annual incident cases of mesothelioma for the years 2006 through to 2015—for males, an average of 97 (range: 69–116); for women, an average of 19 (range: 14–29)—with no apparent time trend. Note that these figures are somewhat lower than the mesothelioma deaths reported recently by Sweden to the WHO (the average of the last three years for all people being 129) [21]. For the latest figures, see the GBD website [22]. All figures are, of course, subject to under-estimation due to lack of detection.

To understand the impact of banning the use of asbestos, epidemiologic research needs to review and monitor experiences of mesothelioma incidence associated with both occupational and non-occupational exposures.

For so long as asbestos is still present in society, research and development programmes are needed for its management as both an occupational and a public-health hazard.

Asbestos is now a widely recognized carcinogen in the case of malignant tumours of the pleura and peritoneum (mesothelioma) and lung cancer. Since mesotheliomas are seen as almost exclusively caused by asbestos, it seems reasonable to expect the legal bans on asbestos use adopted in many Western countries to be related to decreasing mesothelioma incidence and mortality rates.

Järvholm and Burdorf [3] observed in 2015 that the ban in Sweden had prevented an estimated annual 10 mesothelioma cases in men and two in women (below age 57) by 2012. Taking into account lead times of 35 years or more, it is possible to study effects of the ban in birth cohorts from 1980 or later, with analyses to be pursued through the years 2020 to 2030. In a study by Strand in 2011 [23], of 12 mesothelioma cases among engine-room crews in the Norwegian Navy whose starting-times of exposure could be estimated with reasonable certainty, the latency time was found to range from 24 to 48 years (median: 41 years). As noted by Strand, obtaining valid estimates of latency in cohorts exposed to asbestos requires lifelong follow-up.

## 7. Conclusions

The starting point in restricting asbestos use in Sweden was a Communist Party parliamentary motion aimed at total prohibition in 1972. Although the motion failed to gain a majority, it provided grounds for a review authorized by the Government via executive organs. The outcome was a national programme to replace asbestos with less hazardous material whenever this was possible over the years 1973 to 1981. A directive of the National Board of Occupational Safety and Health (NBOSH), enforced from July 1982, included the principle of permitting asbestos use only with the approval of the Board. Reducing the industrial use of asbestos resulted in a dramatic decrease in imported asbestos, up to its near disappearance by the early 1980s. Different types of asbestos were considered. In particular, there was consideration of the relative risks of chrysotile and amphibole by the IARC, since chrysotile (a major Canadian export) was regarded as non-harmful in Canada. Determining factors in restricting asbestos use in Sweden between 1972 and 1985 were: the commitment of Swedish industry to the development of asbestos-free substitute materials and techniques; agreement between industry and labour representatives to make efforts on a tripartite basis with NBOSH; powerful media pressure to restrict all asbestos use; and, the shared cultural instinct of the Nordic countries in Europe to seek consensus when confronting difficult decisions. Asbestos exposure has come to be regarded as not only a work-related, but also an ongoing public-health issue in Sweden, and mesothelioma is now commonly perceived as the critical outcome effect.

The key lessons that other countries still badly affected by asbestos use, or in transition to a ban, might learn from the Swedish experience are that:

- some initial event (in Sweden, a case of whistle-blowing) may be needed to initiate a process towards a reduction or ban;
- sources of exposure need to be identified;
- the media can have great influence;
- the commitment of industry, in particular with regard to finding alternatives to asbestos, is essential, e.g., investing in alternative brake linings in road vehicles;
- reaching consensus between governmental and non-governmental organizations is important;
- sustaining progress towards a ban, which can be quite rapid, involves many different stakeholders whose work needs continuously to be built upon whenever the occasion arises; and
- obstacles, in particular conflicts of interest, can be overcome.

## Appendix A. List of Key Appraisals of the Asbestos Health Hazard in Sweden

Key appraisal events were as follows:

- The NBOSH Asbestos management guideline of 1964—reference made only to the health outcome asbestosis.
- The NBOSH Asbestos management guidelines of 1975 and 1981—references made to the following health outcomes: asbestosis; pleural plaques; lung cancer; mesotheliomas of pleural and abdominal sheath.
- The NBOSH setting of occupational threshold limit values (TLVs) for asbestos fibres 1974–2015 (Note: A TLV value is a time-weighted average representing exposure during a normal working day of 8 h).
- 1974—TLV Guidance no. 100: 2 fibres/mL air for all asbestos forms except crocidolite (permitted for use only following directives of the Factory Inspectorate).
- 1978—TLV Guidance no 100: 1 fibre/mL air for all forms of asbestos, excepting crocidolite (prohibited). Definition of a fibre: “a particle of >5 micrometres in length and a length/diameter ratio greater than 3:1”.
- 1981—NBOSH directives on TLVs 1981:8: 1 fibre/mL air.
  Comment: fibre counting to be carried out using a phase-contrast microscope.
- 1984—NBOSH directives on TLVs 1984:5: 0.5 fibres/mL air.
  Comment: crocidolite prohibited; it may, however, be handled during the demolition of buildings, provided the handling and management are carried out in accordance with the detailed directives of the Factory Inspectorate.
- 1987—NBOSH directives on TLVs 1987:12: 0.2 fibres/mL air (following the EU directive).
- 1990—NBOSH directives on TLVs 1990:13: 0.2 fibres/mL air.
  Comment: Change of particle definition to: “a particle with a length/diameter ratio of at least 5 to 1”.
- 2005—National Environment Agency (earlier NBOSH) directives 2005:17: 0.1 fibres/mL air.
  Comment: Fibre definition changed back to “a length/diameter ratio of at least 3 to 1” (following the EU directive).
